# Visfatin as a predictor of obstructive sleep apnea in atrial fibrillation patients

**DOI:** 10.1007/s11325-020-02025-0

**Published:** 2020-03-13

**Authors:** Anna Szymanska, Anna E. Platek, Janusz Sierdzinski, Filip M. Szymanski

**Affiliations:** 1 Department of Heart Diseases, Medial Centre of Postgraduate Education, Warsaw, Poland; 2 Department of General and Experimental Pathology, Centre for Preclinical Research and Technology, Medical University of Warsaw, Warsaw, Poland; 3 Department of Medical Informatics and Telemedicine, Medical University of Warsaw, Warsaw, Poland; 4 Department of Cardiology, Medical University of Warsaw, Warsaw, Poland

**Keywords:** Obstructive sleep apnea, Biomarkers, Visfatin, Arrhythmia, Sleep disordered breathing

## Abstract

**Background:**

Obstructive sleep apnea (OSA) often coexists with atrial fibrillation (AF) and makes the course of AF worse. The negative impact of OSA on AF may be due to atrial stretch, hypoxia, hypertension, obesity, fibrosis, and inflammation. Several mediators are thought to be responsible for this correlation, among them adipokines such as visfatin. This study aimed to assess the association between visfatin concentrations and OSA in patients with AF.

**Aims:**

This study aimed to assess the association between visfatin concentrations and OSA in AF patients.

**Methods:**

In a tertiary Cardiology Department, hospitalized patients previously diagnosed with AF were enrolled in the study. Diagnosis of OSA was made based on a respiratory polygraphy and patients had blood samples taken for assessment of plasma visfatin concentration.

**Results:**

A total of 266 patients with AF (65% men, age 57.6 ± 10.1) were enrolled, and 121 (45%) were diagnosed with OSA. Patients with OSA had higher visfatin concentrations than those without OSA (2.13 ± 0.17 vs. 1.70 ± 0.21 ng/mL; *p* = 0.04). Patients with mild OSA had visfatin levels equal to 1.77 ± 0.17 ng/mL, moderate OSA 2.38 ± 0.18 ng/mL, and severe OSA 3.55 ± 0.61 ng/mL (*p* for trend = 0.017). Multivariate regression analysis showed that increased visfatin concentrations were associated with the risk of OSA (odds ratio 1.92; 95% confidence interval 1.09–3.40).

**Conclusions:**

Patients with AF who were diagnosed with OSA had significantly higher plasma visfatin levels which increased according to the severity of OSA. Furthermore, multivariate regression analysis identified visfatin concentration over 1.25 ng/mL, male sex, age over 59.1 years, and permanent AF as the factors showing independent correlation with OSA.

## Introduction

Atrial fibrillation (AF) has a highly negative impact on the patient’s prognosis due to its association with an elevated risk of thromboembolic events and stroke [1, 2]. Obstructive sleep apnea (OSA) is one of the conditions that contribute negatively to the AF course. OSA affects at least 2% to 4% of the adult population and the prevalence rates increased over the last 2 decades [3]. OSA is associated with prolonged intermittent hypoxia. It is increasingly recognized and also a major risk factor for cardiovascular disease, including AF. The negative impact of OSA on AF may be due to atrial stretch, hypoxia, hypertension, obesity, fibrosis, or inflammation.

Several mediators are thought to be responsible for this correlation, among them adipokines such as visfatin. Visfatin is also known as nicotinamide phosphoribosyltransferases (NAMPT) and is identical to the pre-B cell colony-enhancing factor (PBEF) [4]. It is synthesized mainly in the visceral fat tissue. Visfatin has an important prognostic utility in patients with cardiovascular disease due to its strong correlation with inflammation, obesity, remodeling, and carcinogenesis. Recent research on visfatin shows also its strong correlation with hypoxia [5]. Various biomarkers are currently being tested as simple and accessible determinants of OSA. The aim of the study was to assess the association between visfatin concentrations and OSA in AF patients.

## Material and methods

The study was an observational study including consecutive patients hospitalized in a Cardiology Department with a primary diagnosis of AF. The regional Ethics Committee approved the study design and protocol, and an informed consent was provided by every participant during the study enrolment. The study included patients aged ≥ 18 years with a confirmed diagnosis of non-valvular AF who were prequalified for heart rhythm control strategy. The study exclusion criteria were overnight respiratory study. Additional comorbidities and cardiovascular risk factors have been diagnosed based on medical history or de novo diagnosis during the current hospitalization. The methodology has been described in detail previously [6]. The diagnosis of AF and its classification into paroxysmal, permanent, or persistent was made base on the current medical practice standards.

In addition to the routine screening and examination, all patients had their blood taken for assessment of visfatin concentration and had an overnight respiratory polygraphy study performed. The study was performed using Embletta MPR devices (Flaga, Reykjavik, Iceland) that are eligible for objective OSA diagnosis confirmation. Scoring of the studies was made manually by a certified sleep medicine specialist, following the American Academy of Sleep Medicine guidelines [7].

Blood samples for biochemical assessment were collected after overnight fasting. Samples were centrifuged for 10 min at 1000 × g for serum and then stored at − 20 °C for assay. Serum visfatin levels were measured with the commercially available sandwich enzyme-linked immunosorbent assay (ELISA, (Cat# K4907-100, BioVision, Milpitas, CA, USA). The method included binding visfatin with absorbed monoclonal antibody to human visfatin. All samples were assessed at the same time in a single certified laboratory. Measuring range was 0.125 to 8 ng/mL, and assay sensitivity was 0.030 ng/mL. All samples were assayed in duplicate, and mean concentration from the 2 assays was included in the analysis. On each analysis plate, a calibration curve was made to assure the proper reading of the assays. The test was done with intra-assay coefficients of variability below 2%. The methodology of biochemical assessment has been described in detail previously [8].

All statistical analyses were performed using SPSS (SPSS version 21, Inc., Chicago, IL) for macOS. Obtained data were tested for normality using the Kolmogorov–Smirnov test. Data showing continuous distribution are presented as mean and 95% confidence intervals (CI), and their comparison was made using the Mann–Whitney test or Student’s *t* test. The categorical variables were compared using the chi-squared or Fisher exact tests. Forward stepwise multivariate logistic regression models were created to identify the independent predictors of OSA. The predictive value of the visfatin concentration was tested by the area under the receiver operator characteristic (ROC) curve.

## Results

Two hundred sixty-six patients were included (65% male, aged 57.6 ± 10.1 years). Arterial hypertension was diagnosed in 72.9% of patients, 8.3% had diabetes, and 10.9% were diagnosed with vascular disease. Paroxysmal AF was observed in 185 (69.5%) patients (Table 1). All patients were also assessed in guideline-recommended risk scores.

**Table 1:** Baseline characteristics of the study population

Parameter	Value ± standard deviation or *n* (%)
Age (years)	57.6 ± 10.1
Systolic blood pressure (mmHg)	131.7 ± 16.7
Diastolic blood pressure (mmHg)	80.7 ± 11.1
Body mass index (kg/m^2^)	29.7 ± 5
AHI (h^−1^)	8.1 ± 10.7
Visfatin (ng/ml)	1.9 ± 2.1
Male sex	173 (65%)
Heart failure	3 (1.1%)
Arterial hypertension	194 (72.9%)
Diabetes mellitus	22 (8.3%)
Stroke or peripheral thromboembolism	23 (8.6%)
Vascular disease	29 (10.9%)
Paroxysmal atrial fibrillation	185 (69.5%)

The respiratory polygraphy revealed OSA in 121 (45.49%) patients. OSA patients were significantly older (59.6 ± 8 vs. 56 ± 11.4 years; *p* = 0.02) with higher body mass index (30.9 ± 5.4 vs. 28.7 ± 4.4 kg/m2; *p* < 0.01) than non-OSA ones. Patients with OSA more often had arterial hypertension (77.7% vs. 69.0%; *p* = 0.03), history of stroke or peripheral thromboembolism (12.4% vs. 5.5%; *p* = 0.03), and vascular disease (14.9% vs. 7.6%; *p* = 0.045) compared to those without the disease.

**Fig. 1:**
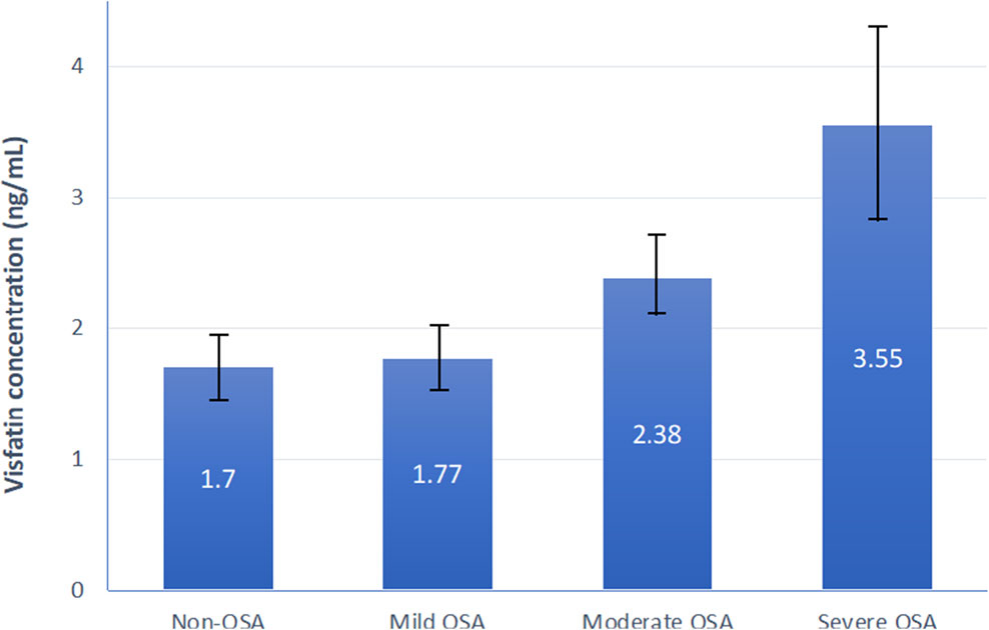
The concentration of visfatin according to the sleep apnea severity

When we analyzed visfatin concentrations, it showed that OSA patients had higher visfatin concentrations than those without OSA (2.13 ± 0.17 vs. 1.70 ± 0.21 ng/mL; *p* = 0.04). Mild OSA patients had visfatin levels equal to 1.77 ± 0.17 ng/mL, moderate OSA 2.38 ± 0.18 ng/mL, and severe OSA 3.55 ± 0.61 ng/mL (*p* for trend = 0.017) (Figure 1). Several other parameters were also positively associated with OSA severity (Table 2).

**Table 2:** Comparison of patients according to the sleep apnea severity

Parameter	Patients without obstructive sleep apnea (*n* = 145)	Mild sleep apnea (*n* = 74)	Moderate sleep apnea (*n* = 35)	Severe sleep apnea (*n* = 12)	*p* for trend
Age (years)	55.9 ± 11.4	59.6 ± 8.5	59.9 ± 7.2	58.6 ± 6.9	0.12
Systolic blood pressure (mmHg)	130.9 ± 16.7	133.2 ± 16.6	132.1 ± 18.3	131.7 ± 15.1	0.88
Diastolic blood pressure (mmHg)	79.7 ± 10.7	82.8 ± 10.7	80.9 ± 12.9	79.6 ± 12.1	0.31
Body mass index (kg/m^2^)	28.7 ± 4.4	30.1 ± 5.5	31.3 ± 4.6	34.4 ± 6.3	0.001
Male sex	88 (60.7%)	55 (74.3%)	24 (68.6%)	6 (50.0%)	0.56
Heart failure	1 (0.7%)	0 (0.0%)	0 (0.0%)	2 (16.7%)	0.008
Arterial hypertension	100 (69.0%)	55 (74.3%)	32 (91.4%)	10 (83.3%)	0.012
Diabetes mellitus	11 (7.6%)	4 (5.4%)	7 (20.0%)	5 (41.7%)	0.001
Stroke or peripheral thromboembolism	8 (5.5%)	9 (12.2%)	4 (11.4%)	2 (16.7%)	0.029
Vascular disease	11 (7.6%)	7 (9.5%)	8 (22.9%)	3 (25.0%)	0.005

Multivariate regression analysis showed that increased visfatin concentrations were associated with the risk of obstructive sleep apnea (odds ratio 1.92; 95% confidence interval 1.09–3.40). Other factors correlating with OSA risk are shown in Table 3. The area under the ROC curve for the visfatin to predict any type of OSA was 0.60; for predicting moderate to severe OSA, it was 0.74 and for severe OSA 0.89.

**Table 3:** Factors associated with an elevated risk of obstructive sleep apnea

Odds ratio estimates			
Effect	Point estimate	95% Wald confidence limits	
Elevated visfatin level^*^	1.916	1.079	3.399
Male sex	2.092	1.147	3.816
Older age^**^	1.062	1.029	1.095
Permanent atrial fibrillation	1.914	1.056	3.470

*Visfatin concentration over 1.25 ng/mL

**Age over 59.1 years

## Discussion

The present study revealed that plasma visfatin concentration (over 1.25 ng/mL) is one of the indicators of OSA. Other factors associated with an elevated risk of OSA are male sex, permanent atrial fibrillation, and age. Moreover, visfatin concentration positively correlates with OSA severity.

Those findings are especially important in AF patients due to the strong association between OSA and AF outcomes. OSA increases the arrhythmia symptoms and progression, worsens the rhythm control strategy outcomes, and is a predictor of increased thromboembolic and cardiovascular risk [9]. Therefore, early diagnosis and treatment of OSA improve patient’s prognosis in terms of cardiovascular health, as well as, for example, outcomes of sinus rhythm restoration procedures.

The underlying mechanisms linking the elevated visfatin concentrations with an increased risk of OSA could be explained by an OSA-caused repeated intermittent hypoxia. Hypoxia causes several changes at both cellular and molecular levels [10]. Hypoxia was shown to be associated with activation of certain genes, including those responsible for the changes in metabolism as well as inflammatory process [11, 12]. Moreover, transient episodes of hypoxia contribute to the development of an increased blood pressure; sympathetic stimulation; increased production of reactive oxygen species; and depletion in the functioning of cardiomyocytes, conductive system, and endothelium [13]. These changes may be responsible for complex remodeling that occurs in OSA patients, especially regarding the cardiac conductive system. It potentially results in arrhythmias such as AF.

Correlation of visfatin with OSA-related hypoxia is probably mediated via HIF1. Studies suggest that visfatin mRNA expression is upregulated in the fat tissue of obesity through the activation of HIF1-alpha pathway due to hypoxia [5]. Moreover, severity and time from the onset of hypoxia (also associated with OSA) correlate with visfatin levels [12]. Therefore, this biomarker may have potential in OSA screening and assessment of OSA-related hypoxemic tissue damage.

The main limitation in the first part of the study was an ambulatory recording used for the sleep evaluation. This type of recordings does not include electroencephalography recording. Because of it, several limitations arise. Hypopneas associated only with arousals are not detected and not included in the data. Sleep time is only approximated and not based on EEG recording. All of this can affect the obtained AHI score [14]. Nevertheless, outpatient RP is widely used and accepted as an alternative for polysomnography, and some major studies are based on this tool [15]. Another limitation of the study is the fact that the visfatin concentration was assessed only once and not reassessed in those scheduled a continuous positive pressure therapy treatment.

In conclusion, the presence of OSA and its severity in AF patients is associated with higher visfatin levels. The multivariate regression analysis identified visfatin concentration over 1.25 ng/mL and such factors as male sex, age over 59.1 years, and permanent atrial fibrillation as a risk factor for OSA. The utility of visfatin assessment for OSA screening requires further studies.
